# Supplementary material for: Are carbon emissions trading and green financial instruments synergistic? -Comprehensive quantitative research based on content analysis
Source: PLoS One. 2024 Mar 7;19(3):e0298601. doi: 10.1371/journal.pone.0298601 (PMC10919601; doi:10.1371/journal.pone.0298601)
Supplement: S1 Data — (ZIP) [file pone.0298601.s001.zip › Data/地级市Z_F工作报告.zip!/╜¡╦╒/╗┤░▓╩╨/└┤╫╘╣1⁄2╓┌║┼í╛┬φ┐╦╩2╛▌═°í┐_2018.docx]

各位代表：

现在，我代表市人民政府向大会作工作报告，请予审议，并请各位政协委员和其他列席同志提出意见。

一、2017年工作回顾

过去的一年，我们在习近平新时代中国特色社会主义思想的指引下，全面贯彻落实中央、省委省政府和市委的决策部署，围绕“两聚一高”“两大目标”，统筹做好改革发展稳定各项工作，经济社会保持平稳健康发展，主要指标增幅继续保持在合理区间。预计全年实现地区生产总值3350亿元、增长7.5%，人均突破1万美元；完成规模以上固定资产投资2800亿元、规模以上工业投资1700亿元，分别增长12%和13%；实现一般公共预算收入230.6亿元，可用财力保持增长；城乡居民人均可支配收入分别达到32800元和15600元，增长8.4%和8.8%。

（一）着力稳增长、调结构，经济运行稳中向好。“稳”的基础不断牢固。坚持“三驾马车”协同拉动，有效投入持续扩大，220个重大项目完成投资1178亿元、增长13.1%，新开工亿元以上工业项目152个，新竣工设备投入超千万元工业项目105个。消费需求保持旺盛，实现社会消费品零售总额1200亿元、增长11%，消费对经济增长的贡献份额达到53%。外贸增长势头强劲，新培育外贸增长点企业120户，完成进出口总额42亿美元、增长20%。“调”的力度日益加大。供给侧结构性改革取得实效，关停化工企业41户，完成取缔“地条钢”非法生产企业和成品油市场整治任务，房地产市场保持总体稳定，制造业贷款增幅苏北第一，银行业金融机构不良贷款率下降0.37个百分点，降低企业运营成本近10亿元，28项补短板任务扎实推进，发展的平衡性和协调性得到增强。“4+2”先进制造业加速集聚，敏安电动汽车、德淮半导体等项目加快推进，国信燃机热电联产、澳洋顺昌二期等项目建成投产，苏淮高新区列入全省首批20家“十三五”先进制造业基地，规模以上工业产值、入库税金、利润分别增长12%、10%和12%。服务业质效提升，增加值增长8.9%，其中“4+3”特色服务业占比77.1%，提高1.1个百分点，金湖水上森林休闲旅游度假区等2个项目列入全国优选旅游项目名录，电子商务交易额增长30%，二类水路开放口岸正式运行，港口货物吞吐量突破亿吨大关。“4＋1”现代农业加快发展，投资超亿元农业产业化项目94个、实现翻番；农村水利完成投入18.2亿元，新建高标准农田35.6万亩，发展稻渔综合种养20万亩，新增高效设施农业7.7万亩，获批中国稻米产业融合发展示范市和全国平安农机示范市。“好”的势头更为强劲。区域创新能力增强，高新技术产业产值增长12%，科技进步贡献率55%。超千户企业实施了技术改造，新培育入库国家高新技术企业71户，新增省两化融合贯标试点企业20户。出台22条人才新政，新建海外引才引智工作站5个，评选认定“533英才工程”学术技术领军人才52名、拔尖人才303名、骨干人才2075名。企业“两站三中心”达到1014个，新增2家国家级研发机构，组建盐化新材料等3个产业技术协同创新联盟。新增国内注册商标3747件、国际注册商标14件，今世缘酒业荣获全国质量奖。淮安高新区获批国家级高新区，淮安国家农业科技园区高分通过验收，涟水现代农业园区创成全国农村创新创业园区，新增施河现代教育体育高端装备、金湖仪器仪表2个国家火炬特色产业基地，经济运行质量效益持续提升。

（二）着力抓改革、促开放，发展活力不断增强。改革红利加快释放。行政审批制度改革深入推进，组建市行政审批局，实施“35745”审改专项行动，实行投资建设项目“四联合”审批，公布737项“不见面”审批事项，营商环境进一步优化，全年新登记私营企业近2万户、个体工商户4.9万户。新型城镇化综合改革稳步实施，“多规合一”试点工作成为全国样板，完善居住证制度配套措施，高沟镇列入省经济发达镇行政管理体制改革试点。土地使用制度改革扎实开展，功能片区土地利用规划加快实施，拓展用地空间3.8万亩，有效开展同一乡镇范围内村庄建设用地布局调整、工矿废弃地复垦利用、洪泽区建设用地“双减量”等试点工作。农村各项改革统筹推进，完成土地承包经营权登记颁证和农村集体建设用地调查，推进承包经营权抵押融资、互换并地和有偿退出等成果应用。侯卫东官场笔记财税金融改革成效明显，有效惠及实体经济、提升财税质态，金融机构本外币贷款增幅全省第一，8户企业登陆资本市场，获批筹建中信银行淮安分行和苏北首家互联网科技小贷公司。公安、环保和审计等领域改革积极推进。对外开放深度拓展。加快台资企业产业转移集聚服务示范区建设，成功举办第十二届台商论坛和台湾淮安周、淮安经贸文化交流合作周等活动，建立淮安昆山台资经济协同发展常态化合作机制，连续第六年获评台商投资“极力推荐城市”，协议台资增长150%，到账台资增长50%。开展日韩、港澳、央企等专题招商活动，新引进亿元以上内资项目210个，其中10亿元以上项目15个；新设立总投资3000万美元以上外资项目40个，其中1亿美元以上项目10个，到账外资11.7亿美元。举办“伟人故里淮安行”系列城市对外交流活动，拓展了对外开放新空间。区域合作成果丰硕。成功承办长三角城市经济协调会第17次市长联席会，牵头成立长三角生态经济专业委员会。宁淮挂钩深度合作规划获批实施，宁淮新兴产业科技园纳入省南北共建园区。签署沿海发展投资基金合伙协议，8个项目列入首批投资计划。援疆、援藏工作取得新成效，与铁岭市对口交流合作全面启动。

（三）着力强功能、重统筹，城乡建设协调推进。规划引领作用不断增强。启动新一轮中心城区控规修编和总体城市设计工作，优化调整28个片区城镇空间规划，编制轨道交通线网、慢行交通系统、海绵城市等规划，颁布实施《淮安市地下管线管理条例》。加强大数据、云计算、地理信息系统在城市规划领域的运用，实现智慧规划与市政务服务大厅的共建共享。中心城市功能稳步提升。投入968亿元，实施793个城建重点项目。竣工金融中心一期工程，全面推进高铁商务区基础设施建设，实施周恩来纪念地及周边环境提升工程，恩来干部学院核心区投入使用，建成龙宫大白鲸嬉水世界，开工建设中国漕运城等重点项目。人居环境日趋优化，实施761个城市环境综合整治项目，改造棚户区186万平方米、老旧小区500万平方米，建成20万吨自来水深度处理工程，提标改造2个主城区污水处理厂，整治26条后街背巷，完成20个小区雨污分流和4条黑臭水体整治，强化城市长效综合管理，开展交通秩序整治等七大专项行动，城市绿色小客厅等3个项目获得省人居环境范例奖。基础设施建设加快推进。连淮扬镇、徐宿淮盐铁路进入全面架梁阶段，淮安机场二期扩建工程主体建成，市区内环高架一期、京杭运河淮海南路大桥等工程快速推进，宿扬高速淮安段、503省道机场连接线、淮海北路古淮河桥、大运河黄码大桥等建成通车。全面完成洪泽湖大堤加固、分淮入沂整治等新一轮治淮流域性工程。智慧交通、智慧教育项目基本建成，人口数据库建成试运行，智慧淮安时空信息云平台、气象灾害预警应急中心开工建设。乡村面貌持续改善。省级重点中心镇规划建设水平不断提升，盱眙龙虾小镇入选首批省级特色小镇，金湖县高桥村入选全省首批特色田园乡村试点村庄，开展洪泽区龟山村等首批市级14个特色田园乡村创建试点。完善村庄环境“五位一体”管护机制，建成农村公路475公里、桥梁88座，镇村公交开通率达到80%以上，顺利完成农村饮水安全巩固提升工程。

（四）着力重生态、优环境，绿色发展迈上台阶。生态建设全面推进。颁布实施淮安市生态文明建设规划，组织修编主体功能区实施规划，编制淮河生态经济带和洪泽湖生态环境保护规划，开展江淮生态经济区、大运河文化带基础性研究，金湖创成全国首批生态文明建设示范县。加快更新绿化树种，成片造林2.83万亩，栽植珍贵乡土树种320万株。绿色转型有力有序。建立能源消耗强度和消费总量双控体系，按照环保信用评价等级对企业实行差别化价格政策，实施30个绿色制造项目。推进市级低碳试点园区建设，加快循环化改造步伐，单位GDP能耗完成省定下降目标。大力推进建筑产业现代化，实现特级资质建筑企业零的突破，4户企业获批省建筑产业现代化示范基地。环境整治成效显现。扎实开展“263”专项行动，扩大高污染禁燃区范围，关停整治10蒸吨以下燃煤小锅炉858台，完成113个挥发性有机物污染综合治理项目，市区PM2.5浓度同比下降6%，空气质量优良天数达到248天，大气质量持续改善。全面建立河长制，完成禁养区畜禽养殖场关闭搬迁任务，有效遏制洪泽湖非法采砂行为，8个国考断面水质和县级以上集中式饮用水源水质全部达标，绿水青山的生态优势日益显现。

（五）着力办实事、惠民生，社会大局安定和谐。公共服务持续优化。认真履行教育职责，修编城乡中小学及幼儿园布局规划，新建61所中小学校和幼儿园，教育现代化创建成绩突出，高考成绩继续保持苏北领先，建成6个省级职教现代化专业群，高等教育和职业教育体系不断健全。综合医改深入推进，成立市级医疗机构药品采购联盟，拓展与省内外高端医疗资源合作，建立5个医疗集团和13个医联体，全面实施分级诊疗制度，提标扩面家庭医生签约服务，淮阴区、淮安区跻身省基层卫生十强县区，有效落实计生惠民政策，创成全国幸福家庭活动示范市。升级改造331个基层综合文化服务中心，颁布实施《淮安市非物质文化遗产保护实施办法》，启动历史建筑普查认定工作，淮剧《留守村长留守鹅》摘得省文华大奖，《淮安年鉴》获全国地方志特等奖。成功举办国际智力运动精英赛、砂板乒乓球世界杯、全国田径大奖赛等赛事，新建一批体育健身公园和乡镇多功能运动场。富民脱贫力度持续加大。认真落实就业创业扶持政策，开展职业技能培训3.4万人，培育新型职业农民4.6万人，实现城镇新增就业9.1万人、再就业5.8万人，扶持城乡自主创业近2万人。创新“阳光扶贫”机制，建立“三个一”帮扶体系，落实“六个精准”措施，加快实施渠北等重点片区扶贫开发五大关键性工程；各级人大代表、政协委员积极发挥自身优势，开展挂钩结对和项目扶贫，经过全市上下共同努力，13万建档立卡低收入人口、45个省市定经济薄弱村实现脱贫。保障水平持续提高。城乡基本社会保险覆盖面达到96%，城乡居民基础养老保险标准提高至每人每月125元。继续提高城乡低保、特困人员供养、残疾人补贴、孤儿养育、医疗救助等标准，组织开展农村留守儿童关爱保护行动，引导支持社会力量参与养老服务机构建设运营。住房公积金中心接入全国异地转移接续平台，住房保障体系健全率96.6%。全面落实优抚安置政策，积极支持军队改革，顺利完成部队转业干部及家属接收安置工作，圆满完成征兵任务。社会治理持续加强。加快法治淮安、平安淮安建设，深入推进“七五”普法，立体化社会治安防控体系逐步完善，创新网格化社会治理机制，刑事、严重暴力和侵财类案件持续下降，非法集资、电信网络诈骗等涉众型经济犯罪得到遏制。深入开展“三进三帮”，扎实推进大排查大整治，认真做好各类社会风险排查管控，有效化解一批矛盾纠纷和信访积案，圆满完成重大安保维稳任务。安全生产形势总体稳定，食品药品安全保障水平持续改善，防震减灾和突发事件应急处置能力不断增强。双拥共建、人民防空、国家安全、民族宗教、机关事务管理、统计、供销、物价、仲裁、档案、广播电视等工作进一步加强，老龄、青少年、妇女儿童、慈善、红十字等各项事业取得了新进步。

（六）着力转作风、提效能，自身建设得到加强。扎实推进“两学一做”学习教育常态化制度化，努力建设人民满意的服务型政府。坚持系统谋划。认真贯彻新发展理念、省“1+3”功能区战略，积极探索生态优先、绿色发展新路子。围绕激发创新活力，出台实施聚力创新20条政策和三年行动计划，着力构建政产学研协同创新体系；围绕促进群众增收，出台实施聚焦富民18条政策，打好产业发展、公共服务、脱贫攻坚组合拳；围绕促进乡村振兴，谋划推进农村相对集中居住；围绕彰显城市特色，立足水资源、做好水文章，大力推进文旅融合发展。坚持真抓实干。落实助推实体经济发展、扩大对外开放“淮十条”等政策措施，集中精力招大引强，实施骨干企业培育“158”工程，完善重大项目月度督查会办、半年督查推进机制，坚持领导干部领办重大项目、服务重点企业。更加注重绩效考评，提高项目质态，谋求不含水分、实实在在的发展。坚持从严治政。旗帜鲜明讲政治，始终把纪律规矩挺在前面。严格落实党风廉政建设责任制，认真执行中央八项规定及《实施细则》精神，规范公共资源交易、政府投资管理和国有资本运作，加强行政监察和审计监督，严肃查处群众身边的不正之风和腐败问题，着力营造风清气正的良好氛围。严格执行重大行政决策程序，建立重大决策出台前向市人大及其常委会报告制度，深化政务公开，主动听取各方意见、接受社会监督，法治政府建设不断加强。

各位代表，以上成绩的取得是省委省政府和市委正确领导的结果，是市人大、市政协和社会各界监督支持的结果，是全市人民团结奋斗的结果。我谨代表市人民政府，向广大干部群众，向人大代表、政协委员和离退休老同志，向各民主党派、工商联、无党派人士和各人民团体，向驻淮部队、武警官兵和部省驻淮单位，向来淮投资创业者，向所有关心支持家乡建设、淮安发展的海内外朋友，表示衷心的感谢并致以崇高的敬意！

在回顾成绩的同时，我们也清醒地看到，当前经济社会发展中还存在不少问题和不足，尤其是发展的不平衡不充分性表现较为突出。从区域来看，县域经济不强，乡镇规模偏小，村庄分布较散，城乡统筹发展水平和城市文明程度有待提升；从产业来看，集聚度还不高，龙头企业带动力不强，中高端产业比重较低，科技创新支撑不够有力，转型发展的压力较大；从满足群众对美好生活的需要来看，富民增收和脱贫攻坚面临新挑战，社会保障、基本公共服务均等化水平有待提升，生态文明建设、社会治理还存在许多薄弱环节；从政府自身来看，职能转变、作风建设和担当意识需要进一步加强。对此，我们将坚持问题导向，采取针对性措施不断加以解决。

二、2018年工作任务

今年是贯彻落实党的十九大精神的开局之年。我们必须不忘初心、牢记使命，抢抓机遇、乘势而上，努力创造无愧于新时代的新业绩。新时代要担当新使命。坚持在全国全省发展大局中，确立对标意识，找准发展标杆，按照“两个一百年”奋斗目标和“两个15年”战略安排，紧扣“两聚一高”“两大目标”，全年工作突出“三个着力”“四大攻坚”“五件大事”，一步一个脚印向既定目标迈进，为2020年全面建成小康社会打牢基础。新时代要厚植新优势。充分发挥国家和省重大战略的带动优势、绿水青山的生态优势、底蕴深厚的文化优势、台资高地的品牌优势、特色产业加速集聚的成长优势和即将进入高铁时代的区位优势，打开新天地，赢得主动权，努力在新一轮区域发展中抢占先机。新时代要应对新挑战。面对宏观调控与资源环境的约束趋紧、稳增长与调结构的双重压力、扩总量与提质效的艰巨任务，我们将以锐意进取的勇气、敢为人先的锐气和蓬勃向上的朝气，勇于开拓创新、攻坚突破，不断推动各项工作迈上新台阶。

今年的政府工作要在习近平新时代中国特色社会主义思想的指引下，全面贯彻党的十九大精神，认真落实中央经济工作会议、省委十三届三次全会和市第七次党代会第二次会议部署，坚持稳中求进总基调，按照高质量发展要求，以供给侧结构性改革为主线，以生态优先、绿色发展为方向，统筹推进稳增长、促改革、调结构、惠民生、优生态、防风险等各项工作，努力实现更高质量、更有效率、更加公平、更可持续的发展。今年全市经济社会发展主要预期目标是：地区生产总值增长7.5%，规模以上工业增加值同口径增长8%左右，规模以上固定资产投资同口径增长9%左右，一般公共预算收入实现同口径增长，社会消费品零售总额增长10.5%左右，研发投入占地区生产总值比重2%左右，城乡居民人均可支配收入分别增长8%左右和8.5%左右，居民消费价格涨幅控制在3%以内，常住人口城镇化率62%，城镇登记失业率控制在4%以内，节能减排确保完成省定任务。围绕上述目标，着重抓好七个方面工作：

（一）主攻重大项目，强力支撑实体经济。坚持以项目为工作第一抓手、发展第一支撑，着力营造项目为王、企业为本的浓烈氛围，大力推进税源建设，不断增强整体实力。

聚精会神招大引强。立足主导产业建链强链补链，充分利用岩盐、凹土等资源优势和各类平台的载体优势，科学编制招商指南和项目名录，分行业、分产业开展专题对接活动，努力引进一批大项目和上下游企业。大力推进委托招商、中介招商、网络招商和基金招商，在省级以上开发园区探索组建投资、建设、金融、人才服务四类集团公司，实行一名县区领导、一个开发平台、一支招商队伍、一套引资政策、一个产业基金的工作机制，进一步强化“一把手”招商，切实提高招商引资成功率。全年新引进亿元以上内资项目210个，其中10亿元以上项目30个；新引进总投资超3000万美元外资项目30个以上、台资项目100个以上，努力实现项目招引新突破。

全力以赴建设项目。强化要素保障，加大服务力度，全力推进市220个重大项目建设。重点抓好200个以上超亿元工业项目，开工建设骏盛新能源电池、哈模模具装备制造等项目，竣工投产德淮半导体、时代芯存和比亚迪智能制造等项目，确保新开工超亿元项目150个、新竣工超亿元项目100个。加快实施200个超5000万元服务业项目，聚力打造西游乐园、中国漕运城和华强方特主题公园等旗舰型项目。大力推进112个超3000万元农业产业化项目，开工建设金茸农业科技、虫草健康农场、全稳农牧科技等龙头项目。突出绩效导向，加强全程督查推进，努力实现项目建设新突破。

动真碰硬优化环境。秉持101%服务精神，打造流动红旗竞赛活动升级版，完善重大项目绿色通道和全程帮办制度，构建“亲”“清”新型政商关系。加大简政放权力度，提高“互联网+政务服务”水平，纵深推进“不见面”审批，让企业少跑腿、好办事。全面推行“双随机一公开”监管，重拳整治不作为、慢作为、乱作为现象，严肃查处“中梗阻”问题，确保行政再提速、效能再提升。落实企业减负政策，加强公共服务平台和要素市场建设，加大金融服务扶持力度，提高产业配套能力，激发和保护企业家创业热情，努力实现营商环境新改善。

（二）聚力产业培育，加快经济转型升级。坚持特色化、规模化、高端化、智能化和绿色化发展方向，一手抓总量扩张，一手抓结构调整，加快构建现代产业发展体系。

集群化发展先进制造业。坚持“一业一策”升级传统产业、做强优势产业、做优特色产业、做大新兴产业，推动产业层次向中高端迈进、供需平衡向高水平跃升，聚力打造“4+2”优势特色产业升级版，力争产值实现两位数增长。推进骨干企业培育工程，落实市县乡三级培育计划，力争销售超亿元企业突破300户、超10亿元企业达到30户，新增规模以上工业企业100户，新培育销售超2000万元的新增长点200个。推动中小企业向“专精特新”发展，新培育小巨人企业10户以上。以“一特三提升”为导向，以垂直整合与横向集聚为重点，推动上下游企业向开发园区集中，打造一批具有影响力和支撑力的产业集群，每个县区在百亿元产业培育上有新突破。

高端化发展特色服务业。加大现代服务业集聚区建设力度，做优“六在淮安”品牌，着力提升“4+3”特色服务业比重。实施“互联网+”20项行动计划，深化与知名电子商务企业合作，拓展电商现代物流园等功能，推动快递业与电商联动发展，加快培育数字经济、创意经济、分享经济和新金融等新业态。规划建设洪泽湖、白马湖等旅游度假区，推动天泉湖创建国家级旅游度假区，精心培育河工小镇、荷韵小镇、韩侯故里等旅游风情小镇，大力发展全域旅游，唱响“水懂我心、自然淮安”的生态旅游品牌。做强核心商圈和重点商贸企业，扶持发展一批淮安“老字号”，推动传统商贸服务业转型升级。启动世界“美食之都”申报工作，办好中国（淮安）国际食品博览会，打造富有地域特色的品牌展会。

融合化发展现代农业。聚焦“三重抓一深化”，大力发展高价值、高颜值农业，以农业供给侧结构性改革为主线，积极对接中国农科院等大院大所，深入推进与省农垦集团、中江国际战略合作，充分发挥各级工商联和商会的桥梁纽带作用，鼓励引导工商资本、社会资本投入农业农村，促进新型经营主体、服务主体和农业品牌建设，加快构建“4＋1”现代农业产业体系、生产体系、经营体系。强化载体支撑，增强农业科技园区、农业产业化示范基地、农产品加工集中区辐射带动能力，推动农业与休闲观光、文化旅游、健康养生等产业深度融合，新招引投资3000万元以上农产品精深加工项目50个，超亿元农业产业化项目10个以上，培育农业全价值链龙头企业10户以上。强化主体培育，新发展家庭农场500个，新增市级以上示范农民合作社100个。落实粮食安全责任制，新增高效设施农业6万亩、渔业面积2万亩。强化品牌引领，依托盱眙龙虾、洪泽湖螃蟹、淮安大米、淮安黑猪、淮安红椒等优质资源，开发公布农产品区域公用品牌统一标识，推动授权主体专业化、公司化运营，完善农产品标准化生产和质量监管追溯体系，增强农业科技服务保障能力，提高国家特色农产品优势区创建实效。

（三）深化改革创新，全面提升开放水平。今年是改革开放40周年，要坚持以改革促开放，以开放倒逼改革，聚焦重点领域和关键环节，不断求突破、上水平、增优势。

全力推动改革攻坚。深入推进“35745”审改专项行动，积极开展相对集中行政许可权改革。加快多证合一、一照一码、证照分离改革步伐，在淮安经济技术开发区实施全链审批赋权改革，在开发园区推行投资建设项目“模拟审批”和全程代办制，在工业品生产许可领域逐步施行“先证后核”审批。扎实推进新型城镇化综合配套改革，抓好金湖全域和9个省级重点中心镇行政管理体制改革试点。深入推进土地使用制度改革，加大五大功能片区规划实施力度，持续开展工业用地弹性出让、先租后让、工矿废弃地复垦利用和洪泽区建设用地“双减量”试点等项改革，探索结余建新指标在县域内调剂使用机制。进一步深化财税金融改革，着力控债务、防风险、强财政、惠民生，筹备成立地方金融监管局，争取更多知名银行来淮设立分支机构，积极发展国有及控股担保机构，推动金融产品和服务模式创新，加大财税金融对实体经济支持力度。深化国企改革，加快转变国有资产监管机构职能，创新国有资本授权经营体制。有序推进农村产权制度改革，进一步稳定承包权、放活经营权、保障收益权，规范和扩大农村产权流转交易市场，让沉睡的资源转化为流动的资本。

全面激发创新创造活力。大力实施创新驱动战略，高新技术产业投资同口径增长9%左右，高新技术产业产值占比26.5%，科技进步贡献率56%，万人发明专利拥有量4件以上。发挥企业创新主体作用，实施“双千”技改升级工程和100项重点技改项目，培育国家高新技术企业100户以上、企业“两站三中心”60家以上。实施“互联网+”“标准化+”“品牌化+”行动，新增两化融合示范试点企业30户、标准化试点项目20个、驰名商标1件以上，推动岩盐、凹土等资源从粗放利用向精深加工、新型衍生品开发转变。加速集聚高端资源要素，新引进1-2个高层次科研机构，评选资助“淮上英才计划”创新创业团队6个、领军人才60名、急需人才400名，力争优势特色产业全部建立产业技术协同创新联盟，促进产业链、创新链、资金链和人才链深度融合。

大力发展开放型经济。积极争取省支持淮安台资集聚示范区建设政策落实，实施“1+4”台资产业发展工程，深化淮台经济文化交流合作，密切与昆山台资经济协作，加快淮台产业融合发展。把发展外向型经济作为促转型、调结构的硬任务，聚焦先进制造业和生产性服务业招引项目，严把外资项目准入关，加强外资项目监管，不断提升利用外资质态，注册外资实际到账实现正增长，其中制造业增长5%、占比40%左右，开工项目到账外资占比70%以上，已落户外资企业增资扩股占比20%以上。促进外贸外经优化升级，新培育外贸进出口企业120户以上，机电和高新技术产品出口占比40%以上，引导企业聚焦“一带一路”，深化境外投资合作，新增境外投资企业10户。加快开发园区体制机制创新，推动开发园区“二次规划”，促进空间优化和资源整合，切实提高集聚能力和产出效益。深化区域合作，积极参与沿海发展联合会运作和沿海“3+3”港口联动，推进“一带一路”江苏沿海发展投资基金项目在淮运作。加快实施宁淮挂钩深度合作规划，承办淮河生态经济带城市协调会暨第一次联席会议，加强与长三角城市交流合作，不断提升援疆、援藏以及与铁岭市对口合作水平。

（四）强化城市建设，不断完善功能品质。坚持精明增长和紧凑城市理念，围绕生态文旅水城定位，大力推进文明城市建设，进一步增强中心城市的综合承载力和辐射带动力。

完善城市布局。完成城市总体规划修编试点，严控城市开发边界，着重在“补白”和“着色”上下功夫，加快形成功能互补、节约高效的空间开发格局。深化“多规合一”平台运用，建立统一的规划审批和监管机制，加大项目选址、建设方案、土地出让的调控力度，促进城市可持续发展。抓好高铁商务区、空港产业园等片区的城市设计，加强地下空间运用研究，加快城市边界地区、城市低效用地和城中村的功能修补、生态修复。强化城市微观空间治理，充分利用历史遗存和地方文化元素，加快景观提升和功能完善，进一步丰富城市公共空间。

提升城市功能。计划投入1015亿元，实施746个重点项目。加快重点片区建设，大力推进高铁枢纽、金融中心等项目，完成智慧淮安基础工程，精心打造淮海中心商业区、水渡口中央商务区等特色商圈，提升生态文旅区核心区功能，促进城市连点成片发展。加快综合交通枢纽建设，推动连淮扬镇、徐宿淮盐铁路主体完工，做好宁淮铁路前期工作，加快市区内环高架一期建设进度，全力争取淮河入海水道二期工程先导段等重大项目开工建设。深入推进人居环境奖城市创建工作，开展15分钟社区生活圈规划研究，统筹布局公共服务设施，扎实推进“厕所革命”，加大老城区停车设施配建与管理力度。完成9000户、100万平方米棚户区改造任务，整治老旧小区130个、改善提升50个。实施控源截污工程，完成17条黑臭水体整治，建成市区备用水源，有序改造居民小区二次供水设施。

彰显城市特色。坚持以绿为韵，个性化、精细化改造提升城市公园和生态绿地，构建绿色步道和慢行系统，形成更多的城市“绿廊”和“绿肺”。坚持以水为脉，加快实施里运河文化长廊、古淮河生态文化廊道等重点项目，加强主城区滨河景观带建设，将生态理念、自然元素、碧水因子注入城市建设各环节，展现城水相依、水绿相映的美丽风貌。坚持以文为魂，加强历史文化名城和非物质文化遗产保护，丰富周恩来纪念地5A级景区内涵，做好大运河文化带项目编报对接工作，打造中国漕运文化核心展示区，不断展现“运河之都”盛景。

强化城市治理。着力构建网格化、精细化、数字化城市管理体系，切实以绣花的功夫管理好城市。加快环卫保洁市场化步伐，推动重要纪念地、重点景区的设施配套和秩序规范。实施道路畅通工程，整治梳理老城区路网，打通大同路、滨河大道，改造29个交通堵点，完善公交线路和换乘体系，优化主次干道交通组织，有效缓解交通拥堵问题。健全抗震、防灾、消防、民防等指挥体系，加强交通运输、危房、燃气等安全监管，强化电梯安全应急和运行维护管理，提升城市安全防护水平。健全城市长效管理机制和工作责任制，加大突出问题通报、约谈和追责问责力度。以更科学的机制、更扎实的举措，开展新一轮全国文明城市创建，引导市民持续提升文明素养。

（五）聚焦乡村振兴，促进城乡融合发展。坚持“四化同步”，加快推进产城一体化和农村相对集中居住，进一步整合资源、优化环境，努力把乡村建设成为美丽幸福家园。

以农村相对集中居住促进镇村建设。坚持以人民为中心的发展思想，统筹部署、分步推进乡镇布局优化工作，今年淮安区、淮阴区、盱眙县和金湖县全面完成相关任务。坚持规划先行，有序推进农村相对集中居住，在五大功能片区和重点中心镇先行推开，金湖县、洪泽区、淮阴区率先全域推进，其他县区选择部分行政村重点突破。推动重点中心镇完善综合功能、加快人口集聚和产城融合，打造辐射带动力强的县域副中心。支持各县区因地制宜试点打造1个具有代表性的特色小镇，提升省市级特色田园乡村试点示范水平，推动各类要素、公共服务向重点镇、重点村集聚，促进产业文化旅游一体发展。

以产业支撑区域经济发展。引导各县区结合自身实际，整合地域相邻、功能相近的产业集聚区、乡镇工业集中区，加快落实一批超10亿元重大项目，重点培育1-2个特色主导产业，形成“一区一主业、园园有特色”的发展格局，努力实现以城带乡、以工促农。鼓励金湖用好生态资源禀赋、争做生态经济示范县，盱眙发挥区位和资源优势、加速融入江北新区，涟水壮大支柱产业、加快城乡统筹步伐、提升城市人口集聚能力；支持清江浦区优化空间、做强区域经济、增强辐射带动能力，淮安区加快特色发展和错位发展、彰显古城风貌、提升总理家乡的窗口形象，洪泽区打响洪泽湖综合生态旅游品牌、加快建设现代化滨湖城市，淮阴区强化国家级高新区和重大产业项目支撑带动作用、提升综合实力。

以共建共享配套基础设施。开工建设白马湖大道等工程，推进348省道、235国道等项目，新改建农村公路300公里、桥梁45座。加快实施黄河故道治理、洪泽湖周边蓄滞洪区安全、中小河流治理等重点项目，全年完成水利投入30亿元以上。加强村庄生活污水治理，开展城乡生活垃圾分类处理，完善乡村环卫收运体系。推进城乡供水一体化建设，加快实现同城同网同质。在优化调整镇村布局的过程中，坚持公共服务做加法，进一步改善群众生产生活条件。

以精准措施推动脱贫攻坚。充分发挥“阳光扶贫”监管系统作用，持续推进特色产业精准扶贫，确保年度新实现10万建档立卡低收入人口、45个省市定经济薄弱村达标脱贫。整合专项扶贫资金和其他涉农资金，加大交通、水利、农业等项目的倾斜力度，推进“一村一品一店”试点示范，加快建设一批富民项目，推动经济薄弱村产业发展和重点片区整体开发。实施家门口就业创业工程，让群众就地就近增收致富。落实农村贫困人口医疗保障政策，加大对因病致贫返贫人群的帮扶力度。

（六）擦亮生态底色，增创绿色发展优势。坚持生态优先、绿色发展，加快生态文明体制改革，做好“生态+”文章，打好污染防治攻坚战，打赢绿水蓝天保卫战。

优化生态空间布局。落实江淮生态经济区发展战略，严把项目准入门槛，调优调绿产业结构，构建以绿色发展为主题的全域生态经济体系，努力打造江淮生态经济区核心区。规划建设生态文旅水城和洪泽湖生态经济区，构建“一区两片四轴”总体空间布局和“两主四副多线”生态安全防护格局。支持洪泽、金湖、盱眙建设省级生态保护引领区，完成国家生态文明先行示范区试点。实施交通要道、河湖廊道和田园村落景观工程，新增成片造林2万亩、省级绿化示范村40个，不断筑牢生态保护屏障。

积极发展生态经济。加快建设绿色园区、绿色工厂、绿色产品和绿色供应链，力争创建绿色工厂2户以上，打造2个国家循环经济示范企业，完成淮安经济技术开发区循环化改造。探索开展近零碳排放示范区创建，积极参与碳排放市场交易，促进绿色低碳循环发展。推进节能减排，优化能源结构，形成6万吨标准煤的节能能力，新增并网光伏发电、风力发电各100兆瓦。推进绿色建筑星级标识认定，加快发展装配式建筑，鼓励发展住宅成品房，完成既有建筑节能改造7万平方米。推进全国低碳交通试点城市建设，加大新能源公交车投放力度，着力构建低碳出行体系。

持续深化专项整治。扎实开展“263”专项行动，推进10蒸吨以上燃煤锅炉整治，完成省定化工企业关停整治任务，有序推进大运河沿线化工企业搬迁。健全大气污染防治和预警机制，加强挥发性有机物治理，实现秸秆机械化还田75%以上，确保空气质量持续改善。深入开展生态河湖行动，抓好河湖“三乱”专项整治，坚决取缔水源地保护区内的违法设施，全面完成古淮河排污治理项目，强化洪泽湖、白马湖水质和湿地保护，确保国考和省考断面水质稳定达标。开展土壤污染状况详查，建立污染地块名录和开发利用负面清单，下大力气整治农业面源污染，全面推进交通干线沿线环境综合整治。加快危险废物集中处置设施建设，严厉查处违法转移固体废物行为。

完善生态监管机制。落实环保管理体制改革方案，加强监测执法能力标准化建设，完善议事协调机制，推进跨区域环境管理。出台生态补偿机制实施方案，加强生态文明建设绩效考核。强化网格化环境监管，实施工业污染源全面达标排放计划，定期公布主要污染物排放超标企业名单，打击恶意违法排污和造假行为。健全以用途管制为主要手段的国土空间保护开发制度，落实国土开发强度、水质优良比例等约束性指标。深入开展节水行动，严厉打击盗取地下水行为，确保通过国家水生态文明建设试点城市验收。

（七）突出共享发展，健全民生保障体系。坚持在发展中保障和改善民生，多谋民生之利，多解民生之忧，努力让人民群众有更多的获得感。

织密公共服务网。大力推进教育现代化建设，优化布局调整，新改扩建20所以上幼儿园，创建30所以上教育现代化先进学校，加强教育高层次人才队伍建设，促进城乡义务教育一体化优质均衡发展，支持大中专院校和科研机构开展产学研协同创新。发展医疗卫生事业，加快实施市公共卫生中心、妇幼保健院新院区、儿童医院等项目，打造一批国家和省级示范基层医疗卫生机构，巩固医联体建设成果，促进家庭医生签约服务提质增效。加强人口战略研究，提升计划生育和妇幼健康服务水平。办好纪念周恩来诞辰120周年系列活动，全面提升恩来干部学院办学水平和服务功能。健全公共文化服务体系，推进文化创意产品开发，提升文化事业与文化产业发展水平。启动中心城区体育设施布局规划修编，做好第十九届省运会参赛备赛工作，办好品牌赛事活动，促进群众体育、竞技体育和体育产业协调发展。实施食品安全战略，深化食品安全城市和放心消费创建，保障群众舌尖上的安全，营造安全有序消费环境。

织密社会保障网。实施全民参保计划，扩大企业养老保险覆盖面，深化机关事业单位养老保险制度改革，推动灵活就业人员和农民工等群体参保，推行新的城乡居民医保实施办法。加快城乡低保标准一体化进程，城乡低保标准分别提高到每人每月580元和520元，符合条件的农村低保对象实现兜底脱贫。加快居家养老服务中心和关爱驿站建设，促进医养融合发展，扩建市老年大学，完善养老服务体系。加快建立多主体供应、多渠道保障、租购并举的住房制度。落实困境儿童分类保障政策，发展残疾人事业，提高社会救助能力。认真做好优抚安置和双拥工作，加强军人荣誉体系建设，加快军民融合深度发展。

织密富民增收网。深入落实各项富民政策，构建长效增收机制，保持居民收入和经济发展同步增长的良好势头。实施积极就业政策，加强职业技能培训，落实援企稳岗措施，畅通农民进城就业通道，新增城镇就业6.5万人、再就业2.6万人，实现农村劳动力转移1.6万人，培育新型职业农民1.5万人。推进“全民创业、淮商崛起”行动，启动第二批省级创业型乡镇、社区、园区创建工作，切实强化创业扶持、优化创业环境。

织密社会安全网。深入推进法治城市和法治县区创建，健全公共法律服务体系，开展普法宣传教育、法治惠民办实事行动。全面深化公安改革，升级社会治安防控体系。完善网格化社会治理机制，实施社会治理大数据平台、“四频一网”等重大项目，提升社会治理智能化水平。落实接访下访机制，推动“阳光信访”升级，有效排查化解社会矛盾，确保信访形势平稳可控。严厉打击非法集资，有效防范和化解金融风险。落实企业主体责任，加大安全隐患排查整治和事故查处追责力度，坚决遏制重特大安全事故发生。完善应急管理和预警信息发布体系，提高突发事件处置水平，营造安定和谐的社会环境。

各位代表，为政之道，民生为本。在全面完成去年十件实事的基础上，今年重点办好提升公共出行能力、优化提升教育资源、提高困难群体保障水平、实现城乡居民医保并轨运行、改善老城区人居环境、保障城乡居民饮水安全、丰富群众文化生活、加快“健康淮安”建设步伐、关爱服务困难老人、实施“雪亮工程”十件实事项目，着力解决好群众最期盼、最迫切的民生问题。

三、全面加强政府自身建设

使命呼唤担当，使命引领未来。进入新时代，政府工作一定要有新气象新作为，更好地肩负起人民的重托，不断开创各项工作的新局面。

科学理政要有新水平。坚持以习近平新时代中国特色社会主义思想武装头脑、指导实践，注重系统性思考谋划、协调性推动落实、整体性发挥功能，不断提升科学跨越发展水平。加强理论学习和实践创新，提升专业能力，弘扬专业精神，以新理念、新思路、新办法战胜挑战、破解难题，持续保持稳中向好的发展势头。树立科学的政绩观，坚持功成不必在我、但功成必定有我，一任接着一任干，多做打基础利长远的好事实事。健全指标体系和考评机制，不搞形象工程、数字攀比，不贪一时之功、一己之名，扎扎实实推动高质量发展。

担当作为要有新建树。锤炼为人民担当、为发展尽责的政治品格，增强工作韧劲和拼劲，在重大事项面前敢于拍板负责，以奋进姿态推动攻坚克难、创新突破。建立落实鼓励激励、容错纠错、能上能下“三项机制”，旗帜鲜明为那些勇于担当、踏实做事、不谋私利的干部撑腰鼓劲。加大行政追责问责力度，让懒政庸政者无以立身，营造机遇面前不迟疑、矛盾面前不回避、困难面前不退缩的干事创业氛围。弘扬立说立行、雷厉风行的过硬作风，定下来的事情说干就干、善作善成，努力交出一份群众认可的成绩单。

依法行政要有新提升。加强法治政府建设，带头尊法学法守法用法，严格规范文明执法。坚持科学决策、民主决策、依法决策，提高行政决策质量和政府治理能力。加强城乡建设管理、生态环境、历史文化保护等重点领域的立法研究，运用法治手段硬化举措、破解难题。完善政务公开、信息发布和舆论引导制度，及时回应社会关切。主动接受人大的法律和工作监督，自觉接受政协民主监督，高度重视社会舆论监督，切实提高政府工作法治化水平。

廉洁从政要有新气象。严守政治纪律和政治规矩，把贯彻中央八项规定精神往深里抓、实里做，坚持不懈纠正“四风”，以上率下放好样子、做好表率。严格落实党风廉政建设责任制，完善权力清单制度，进一步扎牢制度的笼子，真正让权力在阳光下运行。深入落实村级财务阳光监管、村居小微权力制约的各项措施，坚决遏制基层“微腐败”。强化纪律教育和纪律执行，坚决做到知敬畏、存戒惧、守底线，习惯在受监督和约束的环境中工作生活，努力营造风清气正的政务环境。

各位代表，开启新征程，实现新跨越，使命光荣、责任重大。让我们紧密团结在以习近平同志为核心的党中央周围，在省委省政府和市委的正确领导下，创新实干，埋头苦干，奋力谱写新时代崛起江淮精彩篇章！
